# Bericht der Arbeitsgemeinschaft Molekularpathologie der Deutschen Gesellschaft für Pathologie

**DOI:** 10.1007/s00292-024-01378-7

**Published:** 2024-11-11

**Authors:** N. Pfarr, V. Tischler, J. Sperveslage

**Affiliations:** 1 Institut für Pathologie, Technische Universität München, Trogerstraße 18, 81675 München, Deutschland; 2 Institut für Pathologie, Universitätsklinikum Bonn, Venusberg-Campus 1, 53127 Bonn, Deutschland; 3 IMP – Institut für Molekularpathologie, Hohenzollernring 64, 48145 Münster, Deutschland

Die diesjährige Jahrestagung der Deutschen Gesellschaft für Pathologie (DGP) mit der Thematik „Next Generation Pathology“ fand im wunderschönen München statt und wurde in Erinnerung an den verstorbenen Tagungspräsidenten und Institutsleiter Prof. Dr. Wilko Weichert vom Team des Instituts für Pathologie der Technischen Universität geplant. Tagungszentrum war, wie bereits bei der Herbsttagung der AG Molekularpathologie, das Science Center Munich (SCCM) in München-Garching. Die Schwerpunkte der Tagung in diesem Jahr waren „Molekulare Pathologie, Personalisierte Medizin, Computational Pathology, Vergleichende Pathologie“ sowie die AG-Sitzungen zu allen Teilbereichen der Pathologie.

Als größte Arbeitsgruppe der DGP wurden wissenschaftliche Beiträge in 3 Sitzungen am Freitag und Samstag sowie im Rahmen der Posterausstellung präsentiert. Da ein Schwerpunkt der Tagung den Themenpunkt Molekularpathologie umfasste, gab es im Rahmen des Hauptprogramms 3 weitere Sitzungen am Donnerstag, Freitag und Samstag. Die Sitzungen sowie die Posterausstellung der AG wurden vom Kongresspublikum sehr gut und in großer Zahl angenommen.

In der Startsession Molecular Pathology 1 – Data Analysis (Vorsitz: Wolfgang Dietmaier, Regensburg, und Daniel Kazdal, Heidelberg) wurden spannende Vorträge zur bioinformatischen Analyse von Tumorproben (Melanie Börries, Freiburg), zur Varianteninterpretation (Peter Horak, Heidelberg), zum Einsatz von Mutationssignaturen bei der Ganzgenomsequenzierung (Gene C Koh, Cambridge, UK) sowie zu Qualitätsparametern diagnostischer Befunde der Ganzexomsequenzierung aus FFPE-Geweben (Steffen Wolter, Freiburg) präsentiert. Weiter ging es mit der ersten Arbeitssitzung der AG Molekularpathologie (Vorsitz: Nicole Pfarr, München, und Jan Sperveslage, Münster) und dem Update zum Curriculum Molekularpathologie CuMo (Udo Siebolts, Köln), gefolgt von Beiträgen aus Mainz (Julian Rossmanith) sowie der Schweiz (Alex Soltermann, Ittingen). Die zweite Schwerpunktsitzung zu Technologien (Vorsitz: Sebastian Dintner, Augsburg, und Monika Hämmerle, Halle) beschäftigte sich mit dem State of the Art und Ausblick DNA-basierter Techniken in der molekularpathologischen Diagnostik (Sabine Merkelbach-Bruse, Köln), RNA-basierten Techniken (Nicole Pfarr, München) sowie der Proteomik zum besseren Verständnis von Krebsmedikamenten (Bernhard Küster, München), gefolgt von den Präsentationen der Pilotstudie zur Ganzgenomsequenzierung von Formalin-fixierten, Paraffin-eingebetteten (FFPE) Proben (Matthias Hamdorf, Bonn) sowie der epigenomischen und genomischen Analyse primärer und metastatischer Osteosarkome (Albert Roessner, Magdeburg). Die zweite AG-Sitzung (Vorsitz: Udo Siebolts, Köln, und Verena Tischler, Bonn) wurde eröffnet vom Vortrag zur harmonisierten Implementation plasmabasierter *ESR1*-Mutationstestung für Brustkrebspatientinnen (Christoph Jonas, Köln), gefolgt von den Schlussfolgerungen aus dem QuIP-Mikrosatelliteninstabilitäts(MSI)-Ringversuch 2023 für die praktische Anwendung in der MSI-Diagnostik (Wolfgang Dietmaier, Regensburg) und dem Vortrag zur Methylomanalytik im nicht-kleinzelligen Lungenkarzinom (NSCLC) (Theo Kraus, Salzburg). Simon Schallenberg (Berlin) berichtete über KI-basierte, räumlich aufgelöste Zellnischen zur Verbesserung des Stagings bei Patienten mit NSCLC. Elena Lueftl (Regensburg) berichtete über ihre Erfahrungen mit Einzelzell-DNA-Sequenzierung zur Rekonstruktion der klonalen Entwicklung von Tumoren aus FFPE-Proben, gefolgt von Charlotte Kling (München), die die P‑PROFILER-Studie im Kontext homologer Rekombinationsdefizienz im duktalen Adenokarzinom des Pankreas vorstellte. Die 3. Sitzung der AG Molekularpathologie (Vorsitz Nadina Ortiz-Brüchle, Aachen, und Melanie Demes, Frankfurt) beschäftigte sich mit Resistenztestungen bei *Helicobacter pylori* (Michaela Ihle, Köln) sowie der automatisierten Next-Generation-Sequencing(NGS)-Analytik und Befundung (Simon Joke Lambrecht, Freiburg), gefolgt von einem Beitrag von Mohd Yasser (Greifswald) zur NAFLD-mediierten Hepatokarzinogenese. Janna Siemanowski-Hrach (Köln) berichtete zu MSI im Kontext kolorektaler Karzinome und weiterer Entitäten innerhalb des genomweiten NGS. Weiter ging es mit Beiträgen zum molekularen Tumorboard (Uta Matysiak, Freiburg) sowie pankreatischen neuroendokrinen Tumoren (Alexey Kerner, Kiel). In der letzten Sitzung des Schwerpunktprogramms (Vorsitz: Björn Konukiewitz, Kiel, und Nicole Pfarr, München) berichtete Nicola Normanno (Neapel, Italien) über den aktuellen Status und zukünftige Entwicklungen in der Präzisionsonkologie in Europa. Hege Russnes (Oslo, Norwegen) stellte aus pathologischer Sicht die Erfahrungen mit der Implementierung von präziser Krebsdiagnostik in Norwegen vor. Giancarlo Pruneri (Mailand, Italien) trug seine Erfahrungen der Implementierung umfassender genomischer Profilierung in Italien bei. Den Schluss des Zyklus machte Ronny Nienhold (Zürich, Schweiz) mit seinem Bericht zur Validierung von Ganzgenomsequenzierung und ersten Erfahrungen nach der Einführung in der diagnostischen Routine.

In den beiden Postersessions der AG Molekularpathologie wurden insgesamt 22 Poster präsentiert. Aus diesen Sessions wurden 2 Poster von Mitarbeitern der Technischen Universität München für den Posterpreis nominiert. Christina Port aus Garching stellte ihre Arbeit „2NA FISH: a novel spatial transcriptomics tool for therapy decisions based on mRNA diagnostics in the tissue context“ vor und Shounak Chakraborty präsentierte sein Poster mit dem Titel „Classification of cancer types using RNA-seq data from cancer panel TSO500“, wofür er einen der 4 DGP-Posterpreise erhielt.

In der Mitgliederversammlung der AG Molekularpathologie wurden neben dem Status quo der AG Molekularpathologie und deren Entwicklung Informationen zu Tagungen und Kongressen sowie Initiativen aus der AG (wie z. B. das Curriculum Molekularpathologie und der In Vitro Diagnostic Regulation (IVDR) in der Molekularpathologie) vorgestellt. Udo Siebolts wurde nach 7 Jahren Sprechertätigkeit verabschiedet. Wir danken ihm herzlich für sein jahrelanges Engagement und seinen Einsatz im Bereich der Molekularpathologie und freuen uns, dass er uns nun als Mitglied des Beirates der AG Molekularpathologie weiterhin mit Rat und Tat zur Seite stehen wird. In diesem Rahmen wurde Verena Tischler aus Bonn als neues Mitglied des AG-Sprecherteams begrüßt.

Wir blicken auf eine sehr gelungene Jahrestagung der Deutschen Gesellschaft für Pathologie zurück und bedanken uns bei allen an der Organisation Beteiligten, insbesondere dem Leitungsteam der TU München, sowie allen Vortragenden in den Sitzungen sowie den Posterautor*innen der AG Molekularpathologie für ihre qualitativ hochwertigen Beiträge.

Hinweisen möchten wir auf das kommende 14. Herbsttreffen der AG Molekularpathologie im Tagungszentrum Maternushaus in Köln vom 9. bis 10. Dezember 2024 und freuen uns auch darauf, im nächsten Jahr die 108. Jahrestagung der DGP, welche in Leipzig stattfinden wird, als AG Molekularpathologie aktiv mitgestalten zu können.

